# Reduced RhoGDI2 Expression Disrupts Centrosome Functions and Promotes Mitotic Errors

**DOI:** 10.3390/cells14221833

**Published:** 2025-11-20

**Authors:** Mudrika Tripathi, Nancy Garbacki, Jérôme Willems, Gaël Cobraiville, Marianne Fillet, Alain Colige, Christophe F. Deroanne

**Affiliations:** 1 Laboratory of Connective Tissues Biology, GIGA-Cancer, University of Liège, 4000 Liège, Belgium; mtripathi@uliege.be (M.T.); unhapax@gmail.com (N.G.); willems.jrme@gmail.com (J.W.); acolige@uliege.be (A.C.); 2 Laboratory for the Analysis of Medicines (LAM), Department of Pharmacy, CIRM, University of Liege, 4000 Liège, Belgium; gael.cobraiville@chuliege.be (G.C.); marianne.fillet@uliege.be (M.F.)

**Keywords:** RhoGTPases, centrosomes, primary cilia, chromosome congression, immune synapse

## Abstract

RhoGDI2 is a RhoGTPase regulator that has roles in cytoskeleton organization and cell survival, amongst others. It is differentially expressed in many cell types and tissues, including several human cancers, where its expression has been correlated with either good or bad prognosis. To identify the underlying mechanisms, we knocked down its expression in human cancer cell lines. We observed that repression of RhoGDI2 expression, but not that of the closely related RhoGDI1, significantly reduces their proliferation rate. In parallel, RhoGDI2 suppression induces supernumerary centrosomes and inhibits ciliogenesis. As RhoGDIs are regulators of GTPases, we checked whether key RhoGTPases are involved in these effects. We found that silencing RhoA partially rescued the induction of supernumerary centrosomes and ciliary defects observed upon RhoGDI2 silencing. It was previously shown that RhoGDI2 is strongly expressed in immune cells and that there are striking similarities between primary cilia and immune synapses. Based on this knowledge, we silenced RhoGDI2 in NK cells and could demonstrate that this strongly affects their immune synapse-related cancer cell-killing activity. Altogether, these data suggest novel roles for RhoGDI2 in centrosome functions in human cancer and immune synapses in immune cells, which provides an explanation for its reported dual role in cancer.

## 1. Introduction

The small GTPases of the Rho family (RhoGTPases) are well known for their regulatory roles on the actin cytoskeleton and microtubule organization, which affect several main cellular characteristics and functions like morphology, migration, and mitosis. They cycle between active (bound to GTP) and inactive (bound to GDP) forms [1]. Three types of factors regulate this process. Guanine nucleotide exchange factors (GEFs) activate RhoGTPases by allowing the exchange of GDP for GTP, which, consequently, drives the activation of downstream effectors. GTPase-activating proteins (GAPs) stimulate the intrinsic GTPase activity of the RhoGTPases, hence increasing the hydrolysis of GTP into GDP and, therefore, suppressing downstream signaling. Guanine nucleotide dissociation inhibitors (GDIs) extract the RhoGTPases from the membrane and sequester the GDP-bound form of GTPases in the cytosol, protecting them from degradation by enclosing their c-terminal prenyl group but also preventing their activation [2,3,4].

While there are tens of GEFs, GAPs, and downstream effector signaling molecules for RhoGTPases, there are only three RhoGDI [1]. RhoGDI1 (also known as RhoGDIα) is expressed ubiquitously and usually found at high concentrations. It has affinity and interacts with most RhoGTPases. The expression of RhoGDI3 (also known as RhoGDIγ) is usually very low, except in the nervous system. In physiological conditions, RhoGDI2 (also called RhoGDIβ, LyGDI, or D4-GDI) is largely expressed by hematopoietic cells [5], although lower expression can be found in most tissues, suggesting it could have roles extending beyond the hematopoietic lineage. It binds to the same RhoGTPases as RhoGDI1, but with some different affinities [6].

In comparison to RhoGDI1, RhoGDI2 has some specific functions. It was notably reported that it can increase the activity of Rac1 via its interaction with the GEF VAV1 [4] but also by promoting interaction between Rac1 and filaminA [7]. This demonstrates that RhoGDI2 regulates specifically different signaling pathways through mechanisms that are not shared with RhoGDI1. The role of RhoGDI2 in cancer has been evaluated but has led to puzzling observations that it can promote or repress tumor progression and/or metastasis, depending on the cancer types and stage [8,9].

Here, we analyzed the role of RhoGDI2 by repressing its expression in two cancer cell lines. We report that its siRNA-mediated silencing reduces cell proliferation and significantly alters primary cilia formation, a cellular function orchestrated by the centrosome. Silencing of RhoGDI2 significantly increased RhoA activity, and RhoA silencing reversed the inhibition of primary cilia formation observed after RhoGDI2 knockdown. Finally, we observed that RhoGDI2 silencing in natural killer cells inhibited their cell killing activity, a function mediated by the immune synapse, a functional structure exhibiting striking similarities with the primary cilium.

## 2. Materials and Methods

### 2.1. Reagents and Cells

The U-2 OS and MG-63 human osteosarcoma cell lines were obtained from ATCC. The NK-92 natural killer cell line was a kind gift from the Laboratory of Hematology, GIGA-I3, University of Liege. U-2 OS and MG-63 cells were cultured in DMEM (#L0103-500, VWR, Leuven, Belgium) supplemented with 7% fetal bovine serum (FBS) (#10270-106, Gibco, Fisher Scientific, Merelbeke, Belgium). NK-92 cells were cultured in RPMI 1640 (VWR, #L0498-500) supplemented with 10% FBS, 25 mM HEPES, and 5 ng/mL of human recombinant cytokine IL-2 (#124-10CB-29, Proteintech, Manchester, UK). For experiments with co-cultured NK-92 and MG-63 osteosarcoma cells in Incucyte, the cells were co-cultured in Ham’s F-12K medium (Gibco, #21127022) with 7% FBS supplementation. All media were supplemented with penicillin-streptomycin (100 units/mL each) and Fungizone (250 ng/mL), both from Fisher Scientific. Bisbenzimide H 33258 was from Calbiochem (Merck, Overijse, Belgium). Ro-3306 was from SelleckChem (#S7747). Mouse anti-M2 FLAG^®^(#F3165) was from Sigma (Diegem, Belgium). Mouse anti-RhoA (#sc-418) was from Santa Cruz Biotechnology (BioConnect, Huissen, The Netherlands). Mouse anti-Rac1 (23A8) was from Upstate Biotechnology (Sigma). Mouse anti-Cdc42 (610929) was from BD Biosciences (Erembodegem, Belgium). Rabbit anti-ERK1/2 (#M-5670) was from Sigma. The secondary horseradish peroxidase conjugated anti-mouse IgG (#7076) and goat anti-rabbit IgG (#7074) were from Cell Signaling Technology (Bioké, Leiden, The Netherlands). Rabbit and mouse anti-γ-tubulin (#T5192 and #T6557, respectively) and mouse anti- α-tubulin (#T6199) were purchased from Sigma. Rabbit anti-Arl13b (#17711-1-AP) and CoraLite^®^ Plus 488-conjugated Centrin 1 Monoclonal antibody (#CL488-68433) were from Proteintech.

### 2.2. siRNA Transfection and Nucleofection

siRNAs chemically synthesized, desalted, deprotected, and PAGE-purified were from Eurogentec (Liege, Belgium). The sequence of the siRNAs targeting RhoA, Rac1, Cdc42, and RhoGDI1 and of the control siRNA (siScrl) were described and validated previously [10,11]. The sequences of siRNA used for repressing RhoGDI2 expression were as follows: siRhoGDI2(A) (5′-CAGCUGGGUCCCUCUUCAATT-3′ and 5′-UUGAAGAGGGACCCAGCUGTT-3′); siRhoGDI2(B) (5′-GAGCUGGACAGCAAGCUCATT-3′ and 5′-UGAGCUUGCUGUCCAGCUCTT-3′); and siRhoGDI2(C) (5′-GCCUGAAAUACGUUCAGCATT-3′ and 5′-UGCUGAACGUAUUUCAGGCTT-3′). Each pair of oligonucleotides was annealed at a concentration of 20 μm in 50 mM NaCl, 1 mM EDTA, 10 mM Tris-HCl pH 7.5. siRNA transfection was carried out as described previously [12]. Briefly, calcium phosphate-mediated transfection was performed overnight (14–16 h) on subconfluent cells at a final concentration of 20 nM siRNA. Cells were washed twice with PBS and once with complete medium, with this last step being defined as time 0 post-transfection.

siRNA knockdown in NK-92 cells was performed using the Lonza 4D-nucleofector^®^ X unit (AAF-1003X, Lonza, Verviers, Belgium) based on the manufacturer’s recommendation. Briefly, 5 × 10^6^ NK-92 cells were placed in 100 μL electroporation solution with 20 nM siRNA and nucleofected using Nucleofector Program CA-137. Post-nucleofection, NK-92 cells were cultured in complete medium supplemented with 5 ng/mL IL-2 and allowed to recover for 24 h.

### 2.3. Western Blotting

Western blotting was carried out as previously described [13]. Briefly, cells were lysed in SDS-PAGE lysis buffer, and proteins were separated by polyacrylamide gel electrophoresis. Proteins were transferred to a Protran™ nitrocellulose transfer membrane (Perkin Elmer, Mechelen, Belgium). Membranes were then blocked and incubated overnight with diluted primary antibody following the manufacturer’s instructions. Membranes were then washed three times, incubated in the diluted secondary horseradish peroxidase-conjugated antibody for 1 h, and revealed by chemiluminescence using a homemade ECL kit. The membranes were re-probed with an anti-Erk1/2 antibody to obtain protein loading normalization data.

### 2.4. Real-Time Quantitative PCR

Real-time quantitative PCR was carried out as previously described [13]. Briefly, total RNA was isolated using a High Pure RNA Isolation Kit (#11828665001, Roche Molecular Biochemical, Vilvoorde, Belgium) following the manufacturer’s instructions. Five hundred nanograms of total RNA were reverse transcribed using SuperScript III Reverse Transcriptase (Invitrogen, Fisher Scientific). Real-time qPCR was performed in a final volume of 20 μL containing 2 μL of cDNA (corresponding to 10 ng of total RNA), 300 nM of each primer, and 10 μL of the qPCR MasterMix Plus for SYBR^®^ Green (Eurogentec) in a StepOne™ Real-Time PCR system (Applied Biosystems, Halle, Belgium). The results were analyzed with StepOne™ Software v2.1 and normalized to the level of the GAPDH transcript. The primers used for qPCRs are listed in Supplementary Table S1.

### 2.5. Proliferation Assay

Cells were seeded in 24-well costar plates immediately after transfection with siRNA and collected at different time points to determine the DNA content by a fluorimetric technique [14] as previously described [15]. Briefly, cell layers were rinsed three times with saline and homogenized in 600 μL of PBS by sonication (20 s/well). A total of 100 μL of each sample was transferred into a 96-well plate and supplemented with 100 μL of bisbenzimide solution (200 μg/mL bisbenzimide, 4 M NaCl, 20 mM NaH_2_PO_4_, pH 7.4). In each plate, a standard curve of DNA (from 2 μg to 0.03 μg) was included. After plate agitation for 5 min, fluorescence was read in a microplate spectrofluorometer SpectraMax Gemini XS (Molecular Devices, Sunnyvale, CA, USA) with an excitation wavelength of 355 nm and an emission wavelength of 460 nm.

### 2.6. BrdU Incorporation Assay

For these analyses, the BrdUCell ProliferationELISA kit (colorimetric) from Abcam (Amsterdam, The Netherlands) (Ab126556) was used. MG63 cells were seeded in 96-well costar plates immediately after transfection with siRNA. A total of 8 h after seeding, BrdU was added to the culture medium, and the cells were maintained in culture for 16 h. Then, the cells were fixed and processed following the manufacturer protocol.

### 2.7. Immunoprecipitation and Mass Spectrometry Analysis

N-terminal and C-terminal M2-flagged-humanRhoGDI2 sequences were generated by RT-PCR and cloned in pcDNA4/T0. The primers used are listed in Supplementary Table S2. U-2 OS cells were transfected with these vectors and with empty pcDNA4/T0, and resistant clones were selected with Zeocin. 10^7^ U-2 OS ctr or cells expressing either N-terminally or C-terminally flagged RhoGDI2 were lysed. Lysates were immunoprecipitated with anti-M2 FLAG^®^ antibody. The immune complexes were then subjected to mass spectrometry analysis following a previously published procedure [16]. The number of replicates per condition is *n* = 2. The mass spectrometry proteomics data have been deposited to the ProteomeXchange Consortium via the PRIDE [17] partner repository with the dataset identifier PXD069111.

### 2.8. GTPase Activity Assay

The assay was carried out as previously described [12,18]. Briefly, cells were chilled on ice and lysed in a lysis buffer (ice-cold buffer containing 1% Triton X-100, 25 mM HEPES pH 7.3, 150 mM NaCl, 4% glycerol, 0.1 mM AEBSF, 4 μg/mL aprotinin). Lysates were centrifuged for 6 min at 16,000× *g*. Supernatants were immediately frozen in liquid nitrogen and stored at –80 °C until used. An aliquot of each supernatant collected before freezing was denatured in SDS-PAGE lysis buffer to measure the total RhoGTPase content by Western blotting. For pull-down assays, supernatants were incubated for 30 min with 30 μg of GST-CRIB protein containing the Rac binding region or 30 μg of GST-RBD protein containing the RhoA binding region of Rhotekin affinity-linked to glutathione-Sepharose beads. The beads were washed 4 times in lysis buffer and boiled in 60 μL SDS-PAGE lysis buffer.

### 2.9. Immunofluorescence

Cells were seeded on coverslips in a twelve-well plate at 60% confluence. Cells were treated as described in Section 3 for the respective experiments. Post-treatment, the medium was removed, and cells were permeabilized at 37 °C with 0.5% Triton X-100 in 4% paraformaldehyde for 7.5 mins. The permeabilization buffer was aspirated, and cells were washed twice with PBS. Cells were then fixed in 4% paraformaldehyde for 7.5 min, washed twice with PBS, and then blocked for 1 h in PBS supplemented with 5% bovine serum albumin (BSA, Sigma). Immunostainings were performed overnight at 4 °C in PBS+1% BSA (washing buffer) with the indicated antibodies. The coverslips were then washed 3× in washing buffer for 5 min and incubated (for 1 h, in the dark) with fluorescent secondary antibodies diluted in washing buffer (1:1000, goat anti-mouse Alexa-Fluor 488 nm #A21121, or goat anti-rabbit Alexa-Fluor 555 nm #A21429, Fisher Scientific, Merelbeke, Belgium). Cells were washed three times with washing buffer and labelled with a 1:1000 dilution of 4′,6-diamidino-2-phenylindole (DAPI) for 20 mins. The slides were washed once with PBS and once with water and mounted onto coverslips with DAKO fluorescent mounting medium (#S3023, Agilent Technologies, Diegem, Belgium). The coverslips were mounted onto microscope slides, and images were obtained with a Confocal Zeiss LSM880 AiryScan Elyra S1 with a 63x objective.

### 2.10. Tumor Cell Killing Assay

A total of 2000 target cancer cells (MG-63) were seeded per well of a 96-well plate. The next day, the medium was replaced by 50 μL of Ham’s F-12 K medium (to prevent fluorescence background) containing 20 μM of Caspase 3/7 green dye (#4440, Sartorius AG, Göttingen, Germany). Effector immune cells (NK-92) (previously nucleofected with either siScrl, siRhoGDI1, or siRhoGDI2) were then added in a 1:1 ratio (final volume of 200 μL). The plates were then incubated at 37 °C, 5% CO_2_ for 30 mins before being placed into the Incucyte Live-Cell Analysis System (Sartorius AG) for 24 h. We repeated scanning at 1 h intervals.

## 3. Results

### 3.1. RhoGDI2 Silencing Repressed Cell Proliferation

**Figure 1:**
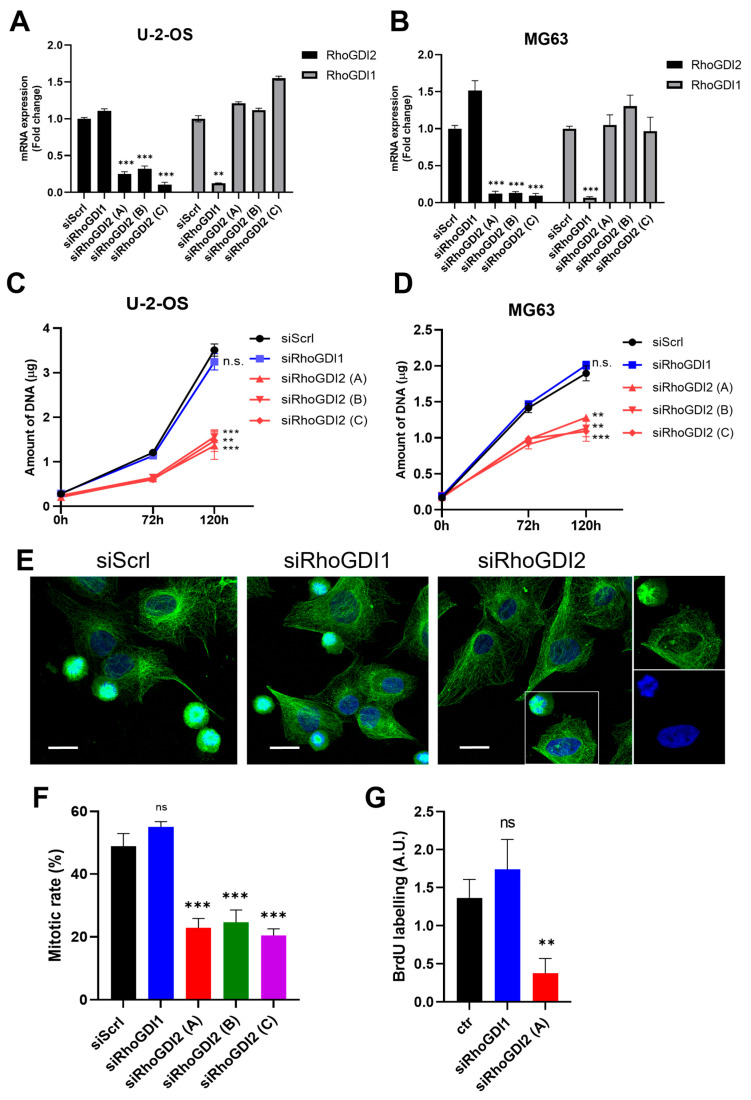
RhoGDI2 silencing represses the proliferation of MG-63 and U-2 OS osteosarcoma cells. Cells were transfected with a control siRNA (siScrl), a siRNA targeting RhoGDI1 (siRhoGDI1), or three different siRNAs specific for RhoGDI2 (siRhoGDI2A, B, or C) and immediately seeded in 6-well plates. After 48 h, cells (U-2 OS in (**A**), MG-63 in (**B**)) were recovered for RT-qPCR analyses of RhoGDI1 and RhoGDI2 expression. A strong and specific silencing was observed, demonstrating the absence of cross-inhibition. Cell proliferation was also evaluated (U-2 OS in (**C**) and MG-63 in (**D**)). After transfection (as in (**A**,**B**)), cells were either collected immediately (0 h) or seeded into 24-well plates for further culture for 72 or 120 h. The DNA content of each well was then measured to assess cell proliferation. In parallel experiments, transfected cells were seeded on cover slips and treated with the CDK1 inhibitor Ro-3306 to arrest cells in the late G2 phase. After 24 h, cultures were extensively washed to remove the inhibitor, and mitosis was evaluated 40 min later by α-tubulin (green) and dapi (blue) staining. Representative images of siScrl, siRhoGDI1, and siRhoGDI2-treated MG63 cells are provided in (**E**). The inserts on the right show separately the green and blue channels. The percentage of cells undergoing mitosis to total cells (mitotic rate) was obtained from 300–450 cells in each condition (**F**). In complementary experiments (**G**), transfected cells were seeded in 96-well plates. A total of 8 h after seeding, BrdU was incorporated for 16 h, and cells were processed following the manufacturer’s protocol to assess BrdU incorporation. The graphs summarize the results of three independent experiments expressed as mean ± s.d. ns, non-significant; ** *p* < 0.01; and *** *p* < 0.001 as determined by ANOVA followed by Bonferroni analysis. Bar = 20 µm.

RhoGDI1 and RhoGDI2 are both expressed in MG-63 and U-2-OS osteosarcoma cells, but at different levels as assessed by RT-qPCR (respectively 22C_T_ and 27C_T_). RhoGDI2 was silenced by RNAi to evaluate its importance for the cell phenotype. As observed by RT-qPCR, RhoGDI2 and RhoGDI1 (used as functional control) expressions were strongly and specifically repressed 48 h after transfection with their respective siRNA in MG-63 and U-2 OS (Figure 1A,B), which lasted for at least 5 days.

The proliferation rate of both cell lines was assessed after 3 and 5 days, as compared to “day 0” (end of siRNA transfection). RhoGDI2 suppression decreased the proliferation rate in both cell lines by approximately twofold as compared to the control (siScrl). Remarkably, the silencing of RhoGDI1 had no significant effect (Figure 1C,D), clearly demonstrating that RhoGDI2 has specific functions that are not shared with RhoGDI1.

To further determine how RhoGDI2 inhibits cell proliferation, siRNA-treated cells were first blocked for 24 h at the G2/M phase (in order to synchronize the cell cycle in the cultures) using the reversible Ro-3366 CDK1 inhibitor. Mitosis was then evaluated by nuclei staining 40 min after extensive washing to remove the inhibitor. We observed approximately 2.5-fold repression of mitosis upon RhoGDI2 inhibition. Once again, no inhibition was observed when RhoGDI1 was silenced, in line with our previous data (Figure 1E,F). This inhibition of proliferation was further confirmed through a complementary approach, by measuring the incorporation of BrdU, which was strongly repressed in RhoGDI2-silenced cells (Figure 1G).

### 3.2. RhoGDI2 Silencing Increases Supernumerary Centrosomes

A co-precipitation experimental model was developed to identify proteins potentially physically associated with RhoGDI2. Cells expressing RhoGDI2 tagged with FLAG^®^ M2 were generated. This tag was placed at either the C- or N-terminus to reduce the probability of missing potential partners specific to the N- or C-terminal end domains of RhoGDI2. Tagged RhoGDI2 was immunoprecipitated with anti-FLAG^®^ M2 antibody, and co-precipitated proteins were identified by mass spectrometry. Several dyneins and centrosome proteins were identified (Table 1), suggesting a role for RhoGDI2 in centrosome functions.

**Figure 2:**
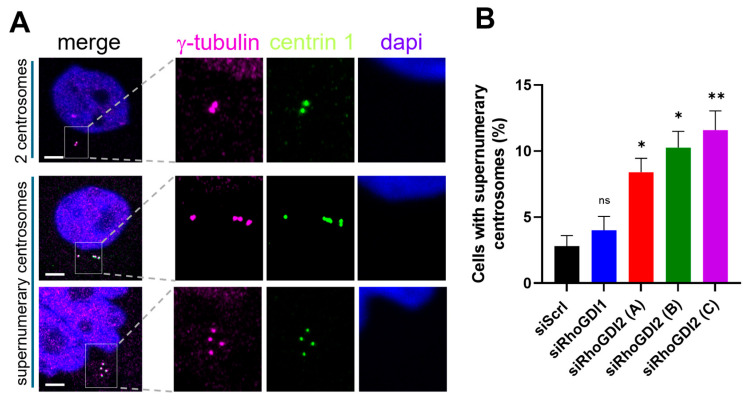
RhoGDI2 silencing increases supernumerary centrosomes. Immediately after transfection (as described in Figure 1), cells were seeded on cover slips and treated with the CDK1 inhibitor Ro-3306 to arrest cells in the late G2 phase. After 24 h, cells were stained for γ-tubulin (pink), centrin 1 (green), and dapi (blue). (**A**) Representative images of cells with 2 centrosomes and supernumerary centrosomes. The percentage of cells with supernumerary centrosomes (>2) in culture after transfection with the different siRNAs is indicated in (**B**). Data were obtained from 250 cells in each condition from three independent experiments, expressed as mean ± s.d. Bar = 5 μm. ns: non-significant; * *p* < 0.05; and ** *p* < 0.01 as determined by ANOVA followed by Bonferroni analysis.

During the cell cycle, centrosomes start duplicating between the G1 phase and the S phase, and this process is highly regulated. Therefore, we investigated whether RhoGDI2 knockdown induced centrosome abnormalities and observed that it led to a significant increase in the occurrence of supernumerary centrosomes (>2 centrosomes) (Figure 2). In some cases, these supernumerary centrosomes are observed in the close vicinity of an enlarged nucleus (compare the nucleus in the second and first examples of supernumerary centrosomes), suggesting aneuploidy. This effect was not observed upon RhoGDI1 knockdown (Figure 2), supporting our previous observations and further documenting that RhoGDI2-mediated regulations can be specific compared to RhoGDI1.

### 3.3. RhoGDI2 Silencing Reduces Ciliogenesis Through RhoA Activation

Centrosomes can form spindle poles during mitosis or lead to the formation of a primary cilium during cell cycle arrest (G0). To evaluate the role of RhoGDI2 on centrosome functions, we tested whether its silencing affects ciliogenesis.

**Figure 3:**
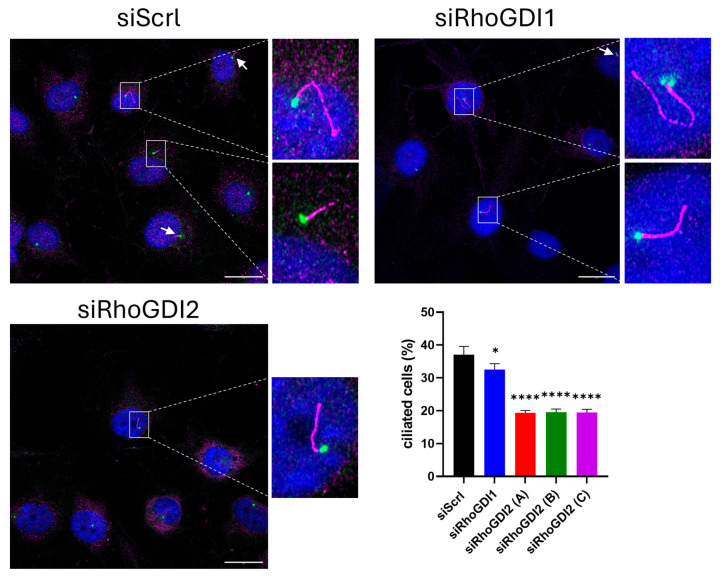
RhoGDI2 silencing reduces ciliogenesis. Immediately after transfection with a control siRNA (siScrl) or with a siRNA targeting RhoGDI1 (siRhoGDI1) or RhoGDI2 (siRhoGDI2A, B, or C), cells were seeded on cover slips and starved for serum for 48 h. They were then treated with the CDK1 inhibitor Ro-3306 for 24 h (to induce the accumulation of cells in the late G2 phase) and co-stained for γ-tubulin (green) and Arl13b (pink). The graphs summarize the results of 750 cells from three independent experiments, expressed as mean ± s.d. Bar = 20 μm. * *p* < 0.05; **** *p* < 0.0001 as determined by ANOVA followed by Bonferroni analysis.

**Figure 4:**
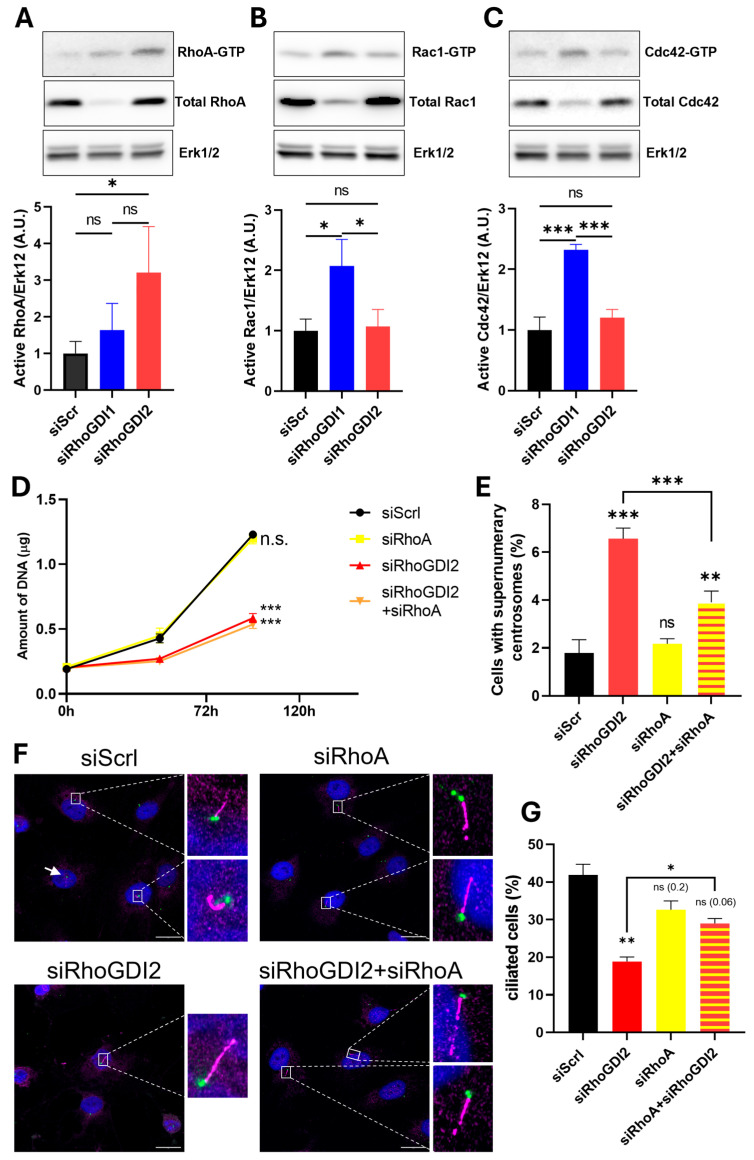
RhoGDI2 silencing increases the activity of RhoA, with no effect on Rac1 or Cdc42 (**A**–**C**). Immediately after transfection (as in Figure 1), cells were rinsed, seeded, and deprived of serum for 48 h. They were then collected and processed to measure, by Western blotting, the total amount and the active fraction (GTP-bound) of RhoA (**A**), Rac1 (**B**), and Cdc42 (**C**). The Erk1/2 abundance was also monitored as loading control in each condition. Quantifications are provided as histograms representing three independent experiments (mean ± s.d). The potential role of RhoA in the RhoGDI2-mediated effects was also evaluated (**D**–**G**). Immediately after transfection (siScrl, siRhoGDI2, siRhoA, siRhoGDI2 + siRhoA), cells were seeded in 24-well plates and collected at different time points for DNA measurements (**D**), or seeded on cover slips, deprived of serum for 48 h and treated with the CDK1 inhibitor Ro-3306 for 24 h to arrest cells in the late G2 phase, before co-staining for γ-tubulin (green) and Arl13b (pink) to label primary cilia (**F**,**G**). RhoA silencing did not rescue the inhibition of proliferation upon RhoGDI2 silencing (**D**) but significantly mitigated the effect on supernumerary centrosomes (**E**) and ciliogenesis (**F**,**G**). The results of each graph are expressed as mean (±s.d.) from three independent experiments. Data in (**E**,**G**) were obtained from 250–300 cells per experiment. Bar = 20 μm. ns: non-significant; * *p* < 0.05; ** *p* < 0.01; and *** *p* < 0.001 as determined by ANOVA followed by Bonferroni analysis.

The knockdown of RhoGDI2 reduced the number of cells with a primary cilium by approximately twofold compared to control, while repression of RhoGDI1 had only a small, albeit significant, effect (Figure 3). The next step was then to investigate the underlying molecular mechanisms responsible for the role of RhoGDI2 in centrosome functions. RhoGDI 1 and 2 interact with multiple RhoGTPases, sometimes with different affinities and effects, and can therefore influence multiple cellular phenotypes through RhoGTPase-regulated pathways. To assess whether RhoGDI2 regulates centrosome functions via the major RhoGTPases, we measured the active (GTP-bound) and total amounts of RhoA, Rac1, and Cdc42 under our different experimental conditions (Figure 4).

The total amounts of RhoA, Rac1, and Cdc42 were greatly reduced upon removal of RhoGDI1, confirming its ability to protect them from degradation [10,19]. Such an effect was not found for RhoGDI2, illustrating once again the existence of different functions for these two proteins, even though they share many sequence similarities (Figure 4A–C). Moreover, we also observed that silencing RhoGDI2 or RhoGDI1 affects the activation of RhoGTPases differently: silencing RhoGDI1 resulted in an increase in GTP-bound Rac1 and Cdc42 but no modification of GTP-bound RhoA, while silencing RhoGDI2 induced an increase in active GTP-bound RhoA but no modification of GTP-bound Rac1 and Cdc42 (Figure 4A–C). This increased RhoA activity suggests its potential involvement in the phenotypical regulations observed following RhoGDI2 silencing. Hence, we then checked whether RhoA participates in the RhoGDI2-mediated regulations (Figure 4D–G).

The RhoA knockdown does not significantly modify the proliferation, supernumerary centrosomes, or the number of ciliated cells. In co-silencing conditions, it does not rescue the inhibition of proliferation induced by RhoGDI2 inhibition (Figure 4D), but it corrects the increased occurrence of supernumerary centrosomes (Figure 4E) and the repression of ciliogenesis induced by RhoGDI2 knockdown (Figure 4F,G), showing that RhoA is part of the RhoGDI2-mediated centrosome regulation. As expected from our data (Figure 4B), the repression of Rac1 in RhoGDI2-silenced cells did not rescue the ciliogenesis phenotype (Supplementary Figure S1).

### 3.4. RhoGDI2 Silencing Reduced Tumor Cell Killing Ability of NK-92 Cells

Having shown that RhoGDI2 repression affects centrosome functions in cancer cells, we wanted to extend these studies to immune cells, where a key function of the centrosome is the formation of the immune synapse (IS) [20] ISs are specialized cell–cell junctions that form between a T or natural killer cell and a target cell, such as a cancer cell, to initiate the killing process. As a functional approach, we tested whether reduced expression of RhoGDI2 affects the ability of NK cells (NK-92) to kill tumor cells, which would indicate changes in IS formation. NK-92 cells were first nucleofected with siRNAs, either control (siScrl) or targeting RhoGDI1 or RhoGDI2. They were then co-cultured with MG-63 osteosarcoma cells pre-loaded with a Caspase 3/7 substrate, which, when cleaved during cancer cell apoptosis, releases fluorescent DNA dye that stains the cell nucleus.

**Figure 5:**
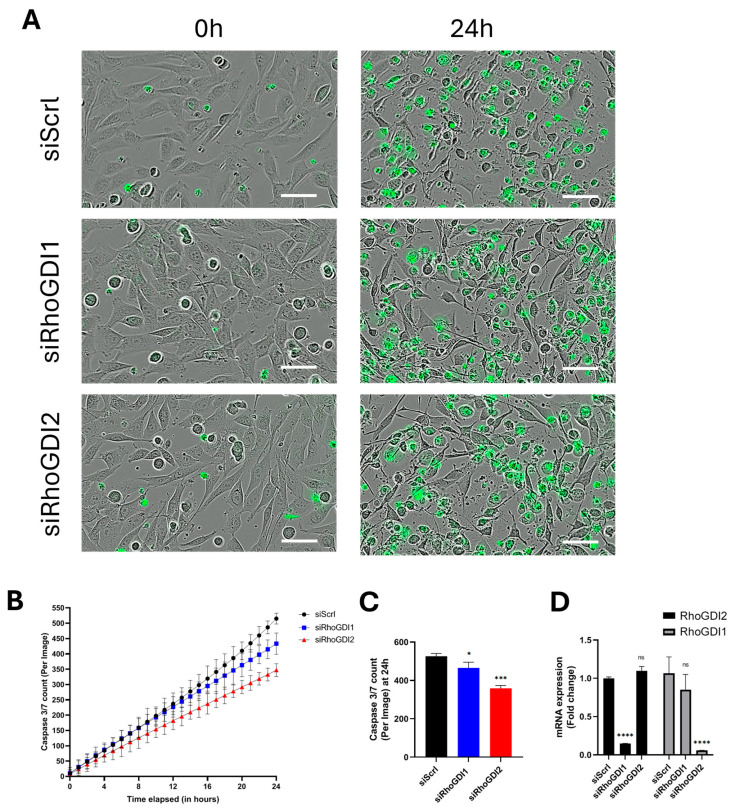
RhoGDI2 silencing reduces tumor cell-killing ability of NK-92 cells. NK-92 cells were nucleofected with a control siRNA (siScrl) or with a siRNA targeting RhoGDI1 (siRhoGDI1) or RhoGDI2 (siRhoGDI2). After 24 h recovery, they were then co-cultured with MG-63 cancer cells loaded with Caspase 3/7 substrates to identify apoptotic cancer cells (in green). Imaging was performed over 24 h (Incucyte Live-Cell Analysis System). (**A**) Representative images of co-cultures at the first (0 h) and last (24 h) time point. (**B**) A 24 h time plot illustrating the killing ability of NK-92 cells (treated with either siScrl, siRhoGDI1, or siRhoGDI2) on MG-63 cancer cells (expressed as green apoptotic cells per field). The killing activity of NK92 cells is strongly affected by RhoGDI2 silencing, even at the beginning of the co-culture. A slight decrease in the killing activity of siRhoGDI1-silenced cells was also observed, but mainly at longer co-culture time. (**C**) Quantification of killing activity at the final time point (24 h). (**D**) Evaluation of the efficiency of RhoGDI 1 and 2 gene silencing in NK-92 cells. The graphs summarize the results of three independent experiments expressed as mean ± s.d. Bar = 50 μm. ns: non-significant; * *p* < 0.05; *** *p* < 0.001; and **** *p* < 0.0001 as determined by ANOVA followed by Bonferroni analysis.

We observed that silencing of RhoGDI2 in NK-92 cells decreased their ability to kill tumor cells by ~30% as compared to control (Figure 5A–C). A smaller, albeit significant, decrease in the tumor-killing ability was also observed upon RhoGDI1 silencing. The efficacy of the gene silencing in NK-92 cells is shown in Figure 5D. These data show that RhoGDI2 controls centrosome function in multiple contexts and cell types.

## 4. Discussion

The Rho GDP dissociation inhibitors 1 (RhoGDI1) and 2 (RhoGDI2) are highly similar; however, they were reported to play differential roles in several types of cancer (review in [8,9]). Although they exert shared functions, notably the regulation of the RhoGTPase activation cycle, in vitro analyses revealed that they have some specific roles in cellular physiology. For instance, in contrast to RhoGDI1, RhoGDI2 was reported to activate Rac1 via its binding to the guanosine exchange factor VAV1 [4]. Importantly, VAV1 could have a role in pancreatic cancer [21,22]. However, these observations can only partly explain the specific contribution of RhoGDI1 and RhoGDI2 to several types of cancers. Hence, our study has been designed to highlight cellular processes relevant to cancer biology that are differentially regulated by RhoGDI2 and RhoGDI1 and that could explain their specific role in vivo. First, we observed that knocking down RhoGDI2, but not RhoGDI1, regulates cell proliferation of various cancer cell types.

To characterize the mechanism involved, we set up an IP/MS (immunoprecipitation followed by identification of proteins by mass spectrometry) to identify proteins that interact with RhoGDI2 and could mediate the observed effects. These analyses revealed the presence of immunoprecipitated proteins related to the centrosome or its function. These proteins are not reported as common contaminants in IP/MS (The CRAPome: a Contaminant Repository for Affinity Purification Mass Spectrometry Data) and were identified with high confidence in terms of peptide identification and protein coverage. They are also consistent with a previous publication showing that RhoGDI2 can be found in association with centrosomes [23]. Surprisingly, Western blot analysis failed to identify these immunoprecipitated proteins. As these interactions between RhoGDI2 and its partners are expected to be highly transient, the efficiency of co-immunoprecipitation is expected to be low. Under these conditions, we speculate that MS analysis is sensitive enough to detect proteins present at low levels, whereas Western blotting is not. As an alternative approach, we then focused on studies that would indirectly confirm the role of RhoGDI2 on centrosome functions.

The centrosome is a non-membrane-bound organelle composed of a daughter and a mother centriole surrounded by a network of electron-dense material known as the pericentriolar matrix. It is the main microtubule-organizing center (MTOC) in the cell and, as such, regulates crucial cellular functions, such as cell shape, polarization, or division [24,25]. The mother centriole is responsible for organizing the cell’s microtubule network, thereby driving the mitotic apparatus to allow the assembly of the mitotic spindle to ensure proper chromosome distribution [26]. It is also the basic element for the formation of primary cilia, a cellular organelle that plays the role of an antenna for various signal transduction, including growth factor signaling but also mechano-transduction or photoreception [27,28]. Alteration of centrosome function or supernumerary centrosomes can affect the faithful distribution of chromosomes between daughter cells and promote chromosome instability, aneuploidy, or mitotic catastrophe. While extra centrosomes are usually viewed as intrinsically pro-tumorigenic, recent investigations suggest that this belief must be adjusted: depending on the context, extra centrosomes can reduce cancer cell fitness and tumorigenesis [29].

We observed that knockdown of RhoGDI2, but not RhoGDI1, inhibited the formation of primary cilia, a microtubule-based organelle that is dependent on centrosomes [28,30]. The primary cilium acts as an ‘antenna’ that integrates various extracellular signals and therefore controls major cellular processes, including proliferation [28]. Ciliogenesis is a process that is strongly regulated by the degree of polymerization of the actin cytoskeleton. Inhibition of actin polymerization promotes ciliogenesis, whereas loss-of-function mutations in ciliopathy genes increase actin stress fibers [31].

The RhoGTPases Cdc42, Rac1, RhoA, and RhoC are involved in actin polymerization, leading to the formation of filopodia, lamellipodia, and stress fibers, respectively, and their activity can be regulated by RhoGDI2. RhoA and RhoC are also involved in centrosome amplification [32]. Therefore, through its association with the centrosome, RhoGDI2 could locally regulate its activity and thus contribute to correct centrosome duplication during the cell cycle. This may explain why we observed that the inhibition of RhoGDI2, but not RhoGDI1, increased the number of cells with supernumerary centrosomes. The increased activation of RhoA, but not Rac1 and Cdc42, upon inhibition of RhoGDI2 suggested that RhoA may be involved in the inhibition of primary cilia formation observed in our model. This was confirmed by an experiment showing that RhoA knockdown significantly decreased the percentage of cells with supernumerary centrosomes and reversed the inhibition of ciliogenesis caused by RhoGDI2 inhibition. By contrast, proliferation inhibition upon RhoGDI2 silencing is not reversed by RhoA knockdown. Proliferation is a multi-step process that involves the coordination of several events in addition to centrosome duplication. RhoGDI2 could affect some of these events independently of RhoA, which would explain why RhoA knockdown is not sufficient to reverse the inhibition of proliferation following RhoGDI2 silencing.

Thus, RhoGDI2 is able to regulate centrosome-dependent cellular functions through local control of RhoA activity, which affects the organization of the actin cytoskeleton. Such local control of RhoGTPase signaling by RhoGDI2 has been suggested in other cellular models, notably in the nucleus of pancreatic b-cells [33]. Spatial regulation of RhoGTPase activity is not limited to RhoGDI2 and has also been described for RhoGDI1, whose local recruitment to cell–cell contacts has been reported to contribute to cell–cell contact maturation in epithelial cells through the regulation of both Rac1 and RhoA [34].

The elimination of target cells by immune cells requires the formation of the immune synapse, an organelle with striking similarities to the primary cilium, including the requirement of the centrosome for its formation [35]. Knowing that RhoGDI2 is highly expressed in immune cells, we then tested whether it regulates their function. We were able to show that inactivation of RhoGDI2 significantly altered the ability of NK cells to kill cancer cells, again highlighting its role in centrosome functions.

Taken together, our observations may explain the dual function of RhoGDI2 in cancer, where it is considered to be either a pro- or anti-cancer factor, as recently reviewed [9]. In cancer cells, it positively regulates centrosome functions and thus their efficient proliferation, while, in immune cells, it favors the formation of the immune synapse, which promotes the killing of cancer cells. Our data clearly indicate that RhoGDI1 and RhoGDI2, although very similar, can perform specific functions. This could be due to different affinities for binding partners [6] but also due to different subcellular locations resulting from differences in their amino acid composition. In particular, the N-terminal parts of their sequences are the most divergent [9], and the analysis of chimeric proteins composed of the N-terminal part of RhoGDI2 and the C-terminal part of RhoGDI1, and vice versa, could help to identify the contribution of these domains to the specific functions of RhoGDI1 and RhoGDI2.

## Figures and Tables

**Table 1:** Dyneins and centrosomal proteins (in blue) as potential RhoGDI2 interactants identified by immunoprecipitation followed by mass spec analysis. RhoGTPases (black) were also identified. RhoGDI2, the immunoprecipitated protein, was detected with the highest confidence and is indicated in red.

Unique Peptides	% Coverage	Score	Protein MW	Accession	Protein Name
22	87.5	276.2	23,044	P52566	Rho GDP-dissociation inhibitor 2
9	34.8	99.7	21,849	P63000	Ras-related C3 botulinum toxin substrate 1
4	18.2	46.2	21,827	P15153	Ras-related C3 botulinum toxin substrate 2
3	18.1	33.7	22,110	P61586	Transforming protein RhoA
4	5.3	28.7	235,152	Q8IZD9	Dedicator of cytokinesis protein 3
4	5.3	26.2	224,984	P11055	Myosin-3
4	2.2	24.3	384,118	A4UGR9	Xin actin-binding repeat-containing protein 2
4	2.4	24.1	524,524	Q96DT5	Dynein heavy chain 11; axonemal
4	7.0	23.8	215,310	Q8TEP8	Centrosomal protein of 192 kDa
4	2.4	23.1	515,918	Q9NYC9	Dynein heavy chain 9; axonemal
3	4.1	22.3	196,273	Q15811	Intersectin-1
3	1.7	21.6	404,166	Q0VDD8	Dynein heavy chain 14; axonemal
3	12.2	21.6	69,618	Q9GZS0	Dynein intermediate chain 2; axonemal
3	5.0	20.4	106,687	O43182	Rho GTPase-activating protein 6

## Data Availability

The mass spectrometry proteomics data are available via ProteomeXchange with identifier PXD069111. Other data will be made available on request.

## Supplementary Materials

The following supporting information can be downloaded at: https://www.mdpi.com/article/10.3390/cells14221833/s1, Figure S1: The repression of ciliogenesis following RhoGDI2 silencing is not rescued by co-silencing of Rac1.; Table S1: Primers used for RTqPCR measurements.; Table S2: Primers used to generate FLAG-RhoGDI2; Uncropped blots.
