# Supplementary material for: Reduced RhoGDI2 Expression Disrupts Centrosome Functions and Promotes Mitotic Errors
Source: Cells. 2025 Nov 20;14(22):1833. doi: 10.3390/cells14221833 (PMC12651903; doi:10.3390/cells14221833)

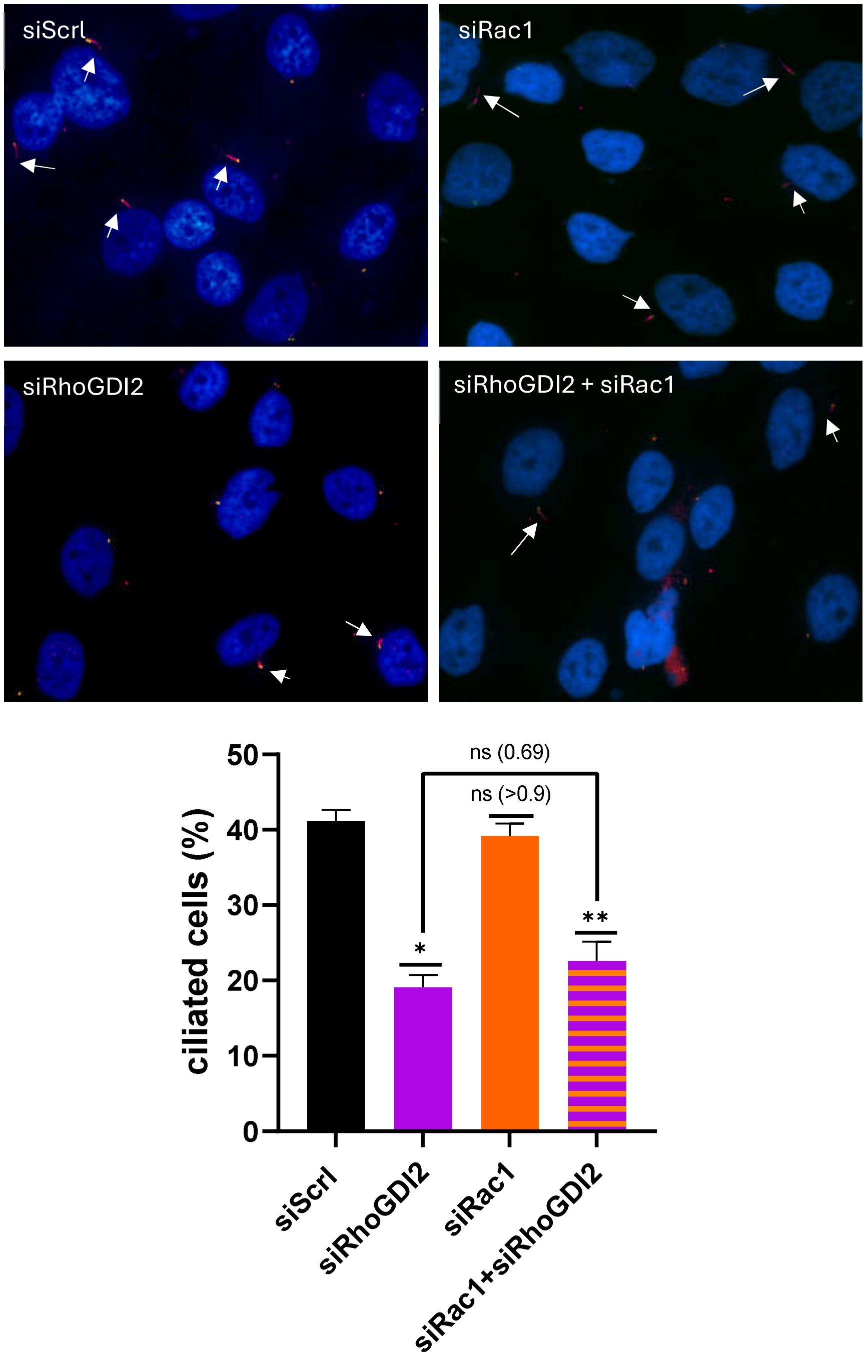

Supplementary Figure 1. The repression of ciliogenesis following RhoGDI2 silencing is not rescued by co-silencing of Rac1. Immediately after transfection with a control siRNA (siScrl), a siRNA targeting RhoGDI2 (siRhoGDI2), a control siRNA and a siRNA targeting Rac1 (siRac1) or with a siRNA targeting Rac1 and a siRNA targeting RhoGDI2 (siRac1+siRhoGDI2), cells were seeded on cover slips and starved for serum for 48 h. They were treated with the CDK1 inhibitor Ro-3306 for 24 h to arrest cells in the late G2 phase and co-stained for γ-tubulin (green) and Arl13b (pink). The results of each graph are expressed as mean (±s.d.) from three independent experiments and shows quantification of ciliated cells in MG-63 cells. Bar = 50 µm. ns non-significant; *P<0.05; **P<0.01; ***P<0.001; ****P<0.0001 as determined by ANOVA followed by Bonferroni analysis.

Supplementary Table 1: Primers used for RTqPCR measurements

Target	Forward primers	Reverse primers
Human RhoGDI2	AAGCCCCAGAGCCACATGT	TCTGTGGTGGAGGCTTATAATTGA
Human RhoGDI1	GCCAAAACTCAAGTCACCTCAGT	AAAGGCAGAGGCAGGACAATAC
Human GAPDH	CCTGGCCAAGGTCATCCATGACA	GGGATGACCTTGCCCACAGCCTT

Supplementary Table 2: Primers used to generate FLAG-RhoGDI2

Amplification product	Forward primers	Reverse primers
N-terminal FLAG RhoGDI2	CACACAGGATCCATGGACTACAAGGACGACGATGACAAGATGACTGAAAAAGCCCCAGA	CACACACTCGAGTCATTCTGTCCACTCCTTCT
C-terminal FLAG RhoGDI2	CACACAGGATCCATGACTGAAAAAGCCCCAGA	CACACACTCGAGTCACTTGTCATCGTCGTCCTTGTAGTCTTCTGTCCACTCCTTCTTAA

UNCROPPED BLOTS

Figure 4A

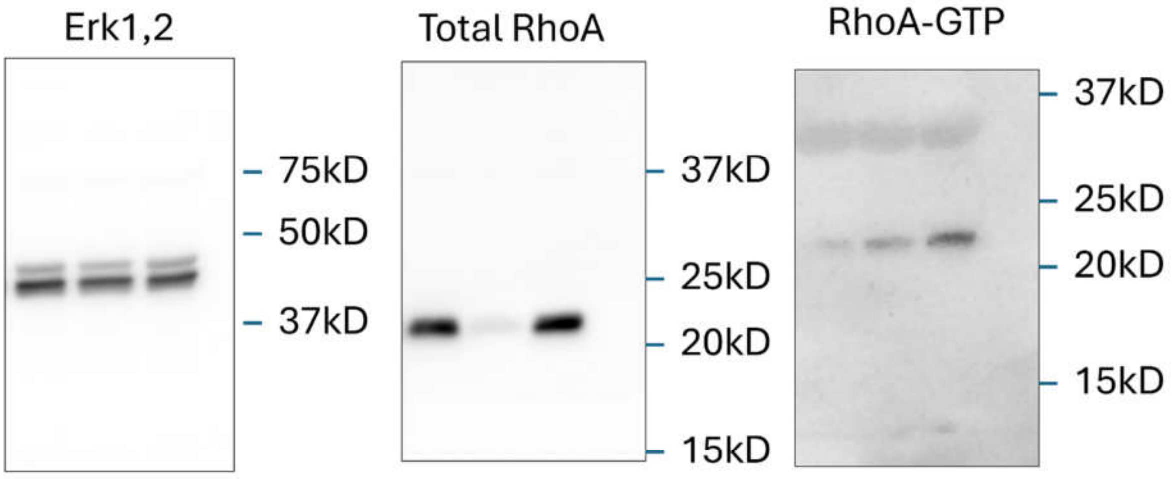

Figure 4B

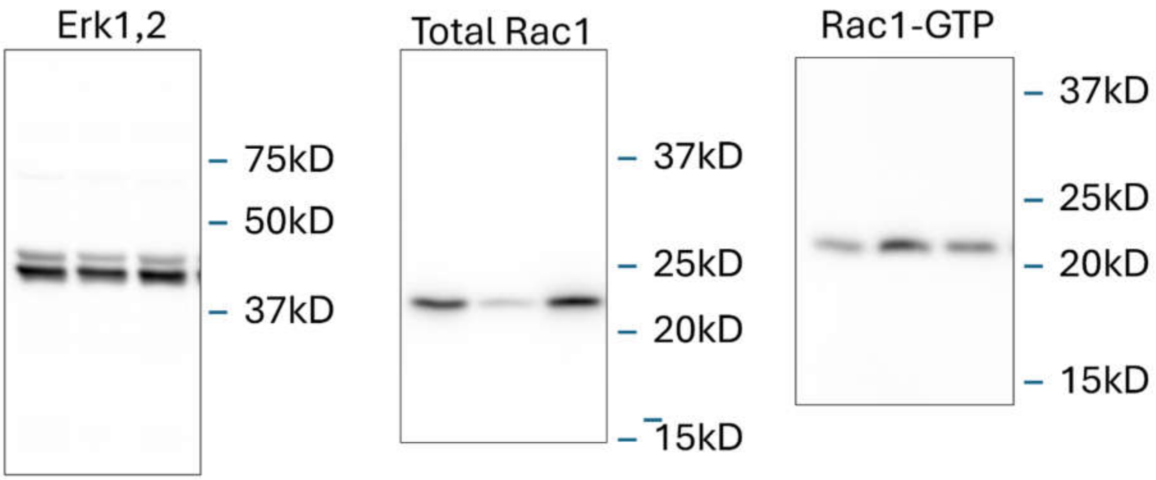

Figure 4C

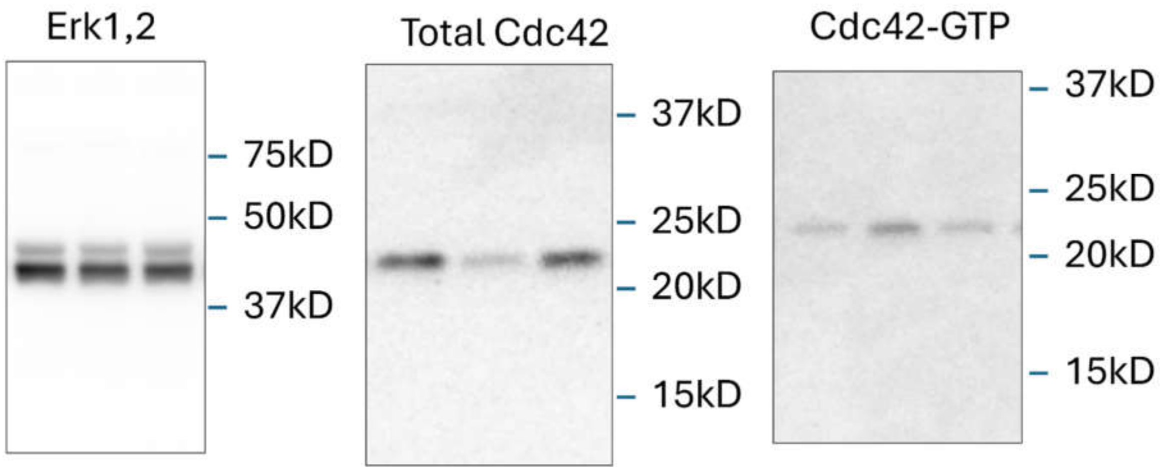

Supplement: Supplementary file 1 [file cells-14-01833-s001.zip › cells-3945829-supplementary.pdf]
